# For Motor Outputs, as for Sensory Inputs, Spike Timing Carries More Information than Spike Rate

**DOI:** 10.1371/journal.pbio.1002019

**Published:** 2014-12-09

**Authors:** Richard Robinson

**Affiliations:** Freelance Science Writer, Sherborn, Massachusetts, United States of America

One of the fundamental problems in neuroscience is understanding the relationship between neural activity and the behavior it produces. In the study of neurons that control motor systems, that output has typically been quantified in terms of firing rate, measured as the number of spikes per unit time. By contrast, for sensory systems, the firing pattern—how those spikes are temporally distributed across that unit of time—has emerged as critical to understanding the encoding of sensory information. Two different temporal patterns, even though they share the same firing rate, can encode two different sensory inputs.

New work by Claire Tang, Samuel Sober, and colleagues in this issue of *PLOS Biology* indicates that, just as in sensory systems, the motor output that controls bird song is dictated not only by firing rate but also by firing pattern and the information inherent in those patterns greatly exceeds the information available in simple spike counts.

The authors recorded the song of the adult Bengalese finch while simultaneously recording neuronal outputs from the robust nucleus of the arcopallium (RA), a premotor cortical region that organizes vocal patterns, whose neurons synapse onto motor neurons controlling the vocal muscles.

**Figure 1:**
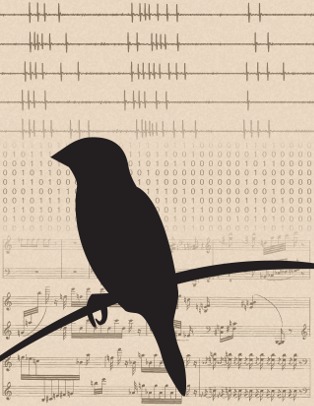
Millisecond-scale motor encoding in the songbird brain. Patterns of electrical activity in neurons (voltage traces, top) control skilled behaviors. Representing neural activity as binary “words” (middle) reveals how the brains of songbirds use these electrical patterns to produce vocal output. The precise timing of neural signals appears to be crucial in controlling the elaborate vocal patterns that have fascinated human listeners for centuries (bottom, excerpt from Olivier Messaien's *Oiseaux exotiques*, which was based on Messaien's transcriptions of birdsong). *Image credit: Sam Sober*.

The basic unit of a bird's song is a note; one or more notes are combined to make a syllable, syllables are repeated to form a motif, and motifs together make a song. Iterations of each song syllable were classified into “behavioral groups,” based on the acoustic properties of pitch, amplitude, and “spectral entropy,” which is the measure of noise versus tonality of a given sound. Together, these three features capture most of the variation among the syllables produced by the finch. Electrodes in the RA allowed the authors to record spike trains that immediately preceded a syllable, thus enabling them to compare the firing parameters with the characteristics of the song it produced (Figure 1).

With the spike trains and acoustic analysis in hand, they next asked whether the firing rate (“spike counts”) or firing pattern (“spike timing”) of RA output was the best predictor of the behavioral group a given syllable would fall into. This analysis involves asking what the “cost” is of transforming one train into another, based on assigning individual costs to actions such as advancing or delaying a spike by a millisecond or adding or subtracting a spike. The final cost is the measure of dissimilarity, or “distance,” between the two trains. These distances are then used to independently classify the syllables they give rise to into behavioral groups, and those classifications are compared to the ones from the acoustic analysis.

If only spike counts are important in determining vocal characteristics, then any cost assigned to advancing or delaying a spike would increase the distance between two trains without improving the accuracy of the classifier. On the other hand, if spike timing was important, then the cost of that advance or delay would be compensated for by an improvement in the performance of the classifier. The authors' analysis indicated that consideration of spike timing led to better prediction of vocal characteristics three to four times as often as consideration of spike count.

Next, the authors performed a second type of analysis, which examined the predictive information present in finer and finer timescale resolutions, from 40 milliseconds down to 1 millisecond, the shortest interval they could reliably measure. They found that as the temporal resolution increased, so did the information present, suggesting, in the authors' words, “that small differences in spike timing can significantly influence motor output.” Indeed, the information present at that resolution was about ten times what was available from the spike rate alone.

The results of these analyses have several implications. Most directly, restricting neural analysis to spike timing will likely significantly underestimate the information output of motor systems. Furthermore, while the spike trains analyzed here were from single neurons, the authors note that even higher-order information may be present in the coordinated output of multiple neurons. Finally, whether time-coded sensory inputs feed directly into time-coded motor outputs will require further investigation, but the demonstration of the existence of such outputs clears the way for those investigations.

**Tang C, Chehayeb D, Srivastava K, Nemenman I, Sober SJ (2014) Millisecond-Scale Motor Encoding in a Cortical Vocal Area.** doi:10.1371/journal.pbio.1002018

